# The proteolytic activity of *Listeria monocytogenes* HtrA

**DOI:** 10.1186/s12866-019-1633-1

**Published:** 2019-11-14

**Authors:** Carmen M. Abfalter, Sabine Bernegger, Miroslaw Jarzab, Gernot Posselt, Karthe Ponnuraj, Silja Wessler

**Affiliations:** 1 Department of Biosciences, University of Salzburg, Billroth Str. 11, A-5020 Salzburg, Austria; 2 Centre of Advanced Study in Crystallography and Biophysics, University of Madras, Guindy Campus, Chennai, India

**Keywords:** *Listeria monocytogenes*, HtrA, Protease

## Abstract

**Background:**

High temperature requirement A (HtrA) is a widely expressed chaperone and serine protease in bacteria. HtrA proteases assemble and hydrolyze misfolded proteins to enhance bacterial survival under stress conditions. *Listeria monocytogenes* (*L. monocytogenes*) is a foodborne pathogen that induces listeriosis in humans. In previous studies, it was shown that deletion of *htrA* in the genome of *L. monocytogenes* increased the susceptibility to cellular stress and attenuated virulence. However, expression and protease activity of listerial HtrA (LmHtrA) were never analyzed in detail.

**Results:**

In this study, we cloned LmHtrA wildtype (LmHtrA^wt^) and generated a proteolytic inactive LmHtrA^SA^ mutant. Recombinant LmHtrA^wt^ and LmHtrA^SA^ were purified and the proteolytic activity was analyzed in casein zymography and in vitro cleavage assays. LmHtrA activity could be efficiently blocked by a small molecule inhibitor targeting bacterial HtrA proteases. The expression of LmHtrA was enhanced in the stationary growth phase of *L. monocytogenes* and significantly contributed to bacterial survival at high temperatures.

**Conclusions:**

Our data show that LmHtrA is a highly active caseinolytic protease and provide a deeper insight into the function and mechanism, which could lead to medical and biotechnological applications in the future.

## Background

*Listeria monocytogenes* (*L. monocytogenes*) is a Gram-positive pathogen that can induce listeriosis, which is a rare fatal foodborne disease exhibiting a mortality rate of approximately 20–30%. *L. monocytogenes* occurs ubiquitously in the environment and can be transmitted via food since it is highly resistant to environmental conditions and food processing [1, 2]. Hence, *L. monocytogenes* is a major concern, both for the food industry and health organizations. In the human body it can cross the intestinal barrier, the blood-brain barrier, and the fetoplacental barrier, and thus can infect organs such as the brain or uterus. Hence, *L. monocytogenes* causes severe life-threatening infections like meningitis, encephalitis, spontaneous abortion, or miscarriage [3].

As a facultative intracellular pathogen *L. monocytogenes* can actively invade and multiply within macrophages and nonphagocytic epithelial cells from where it can spread to neighboring host cells [3]. The bacterial surface molecules internalin A (InlA) and internalin B (InlB) establish the first contact to the host cells followed by internalization of *L. monocytogenes* within a membrane-enclosed compartment [4]. Release from the internalization vacuole requires the pore-forming toxin listeriolysin O (LLO) and the two phospholipases PlcA and PlcB allowing *L. monocytogenes* cytoplasmic localization [3]. In addition, *L. monocytogenes* expresses many factors that sense hostile changes during infection, which are pivotal to promote successful infection. Several years ago, the chaperone and serine protease high temperature requirement A (HtrA) was identified as an important factor in stress tolerance and virulence of *L. monocytogenes*. Lack of HtrA expression led to an attenuated growth under stress conditions, such as elevated temperatures, acidic pH, or oxidative stress [5, 6]*. In a murine L. monocytogenes infection model, a significant lower number of htrA-negative pathogens was re-isolated from spleen compared to the wildtype strain suggesting* an important role for HtrA in listerial growth and survival during infection [6]. In addition, a *L. monocytogenes ΔhtrA* deletion mutant showed a severe defect in biofilm formation and an attenuated virulence in mice [7]. A recent study suggested a putative function of HtrA in listerial replication in infected host cells and underlined the importance of HtrA for tolerating the changing environment in the infection process [8].

The implication of HtrA in bacterial virulence has been demonstrated in many pathogens, including *Helicobacter pylori*, *Campylobacter jejuni*, *Borrelia burgdorferi*, etc. [9–12]. The principal function of HtrA is associated with protein quality control and the degradation of misfolded proteins to enhance bacterial fitness under stress conditions. In addition, HtrA is involved in the processing of other important bacterial virulence factors and cleaves cell surface proteins on host cells indicating multiple mechanisms to promote bacterial pathogenesis [9–12].

The domain structure of HtrA proteases differs between Gram-positive and Gram-negative bacteria. *Escherichia coli* (*E. coli*) expresses the three HtrA homologues HtrA/DegP, DegQ, and DegS. DegP and DegQ contain a cleavable N-terminal signal peptide, which is responsible for periplasmic localization. The signal peptide is followed by the serine protease domain harboring the catalytic triad histidine, aspartic acid and serine. Further, DegP and DegQ harbor two PDZ (Postsynaptic density of 95 kDa, Discs large and Zonula occludens) domains mediating protein-protein interactions, substrate recognition and binding. In comparison to *E. coli*, HtrA proteases in Gram-positive bacteria often harbor a transmembrane domain instead of a signal peptide and only a single PDZ domain [13, 14].

HtrA protease regulation in response to binding to misfolded or native substrates was mainly investigated in *E. coli* in which DegP forms trimers, hexamers, dodecamers, and finally 24-mers [14, 15]. It has been suggested that upon binding to misfolded proteins, DegP switches from proteolytic inactive hexamers to active 12-mers and 24-mers [14]. Several substrates for HtrA have been described, including misfolded proteins (e.g. maltose binding protein, alkaline phosphatase A, α-amylase, etc.) and native proteins (e.g. acylated precursor of colicin A lysis protein, CpxP, E-cadherin, etc.) [13].

Although the implication of HtrA in *L. monocytogenes* pathogenesis has been consistently reported, the regulation and activity of listerial HtrA are completely unknown. In this study, we selected the well-established *L. monocytogenes* references strains 10403 s and EGD and demonstrated enhanced expression of endogenous HtrA in the stationary growth phase. Active recombinant *L. monocytogenes* HtrA was produced and the proteolytic activity under elevated temperatures was demonstrated. Finally, a small molecule inhibitor targeting bacterial HtrA proteases efficiently blocked *L. monocytogenes* HtrA activity.

## Results

**Fig. 1:**
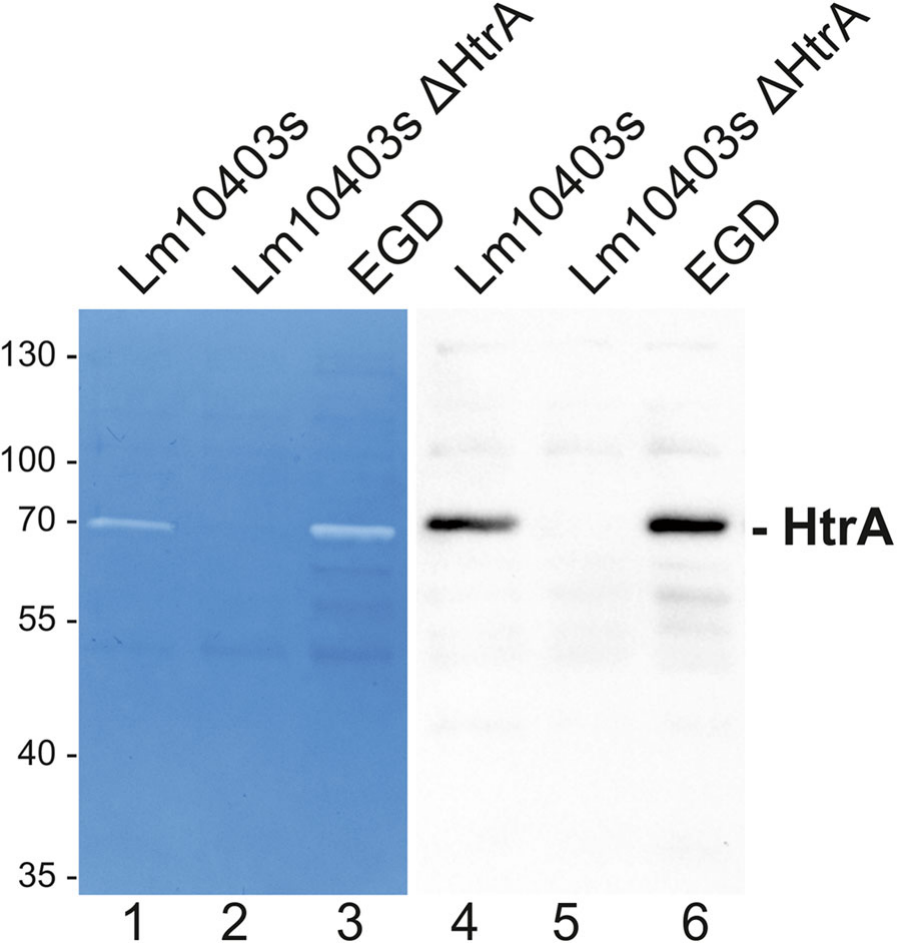
*L. monocytogenes* expresses caseinolytic active HtrA. Equal amounts of protein lysates of *L. monocytogenes 10403 s, L. monocytogenes ∆htrA, and L. monocytogenes* EGD were analyzed by casein zymography (left panel) to detect caseinolytic active proteases and Western blotting (right panel) to detect HtrA expression. The zymograms have been repeated four times and the HtrA detection by Western blotting has been repeated at least three times using lysates from independent experiments

Although the presence of HtrA has closely been correlated with *L. monocytogenes* pathogenesis [5–7], HtrA protein expression and activity was never shown so far. Bacterial lysates of Lm10403s wildtype, its isogenic *ΔhtrA* mutant, and *L. monocytogenes* EGD wildtype were analyzed by casein zymography to detect proteolytic activities. In both wildtype strains, a caseinolytic activity was detected, which was absent in the *ΔhtrA* deletion mutant (Fig. 1, lane 1–3). The existence of HtrA proteins within the proteolytic protein band was verified by mass spectrometry from a preparative zymogram (data not shown). Correspondingly, the expression of the HtrA protein in listerial wildtype strains was demonstrated by Western blot analyses using a polyclonal anti-LmHtrA antibody (Fig. 1, lanes 4–6).

**Fig. 2:**
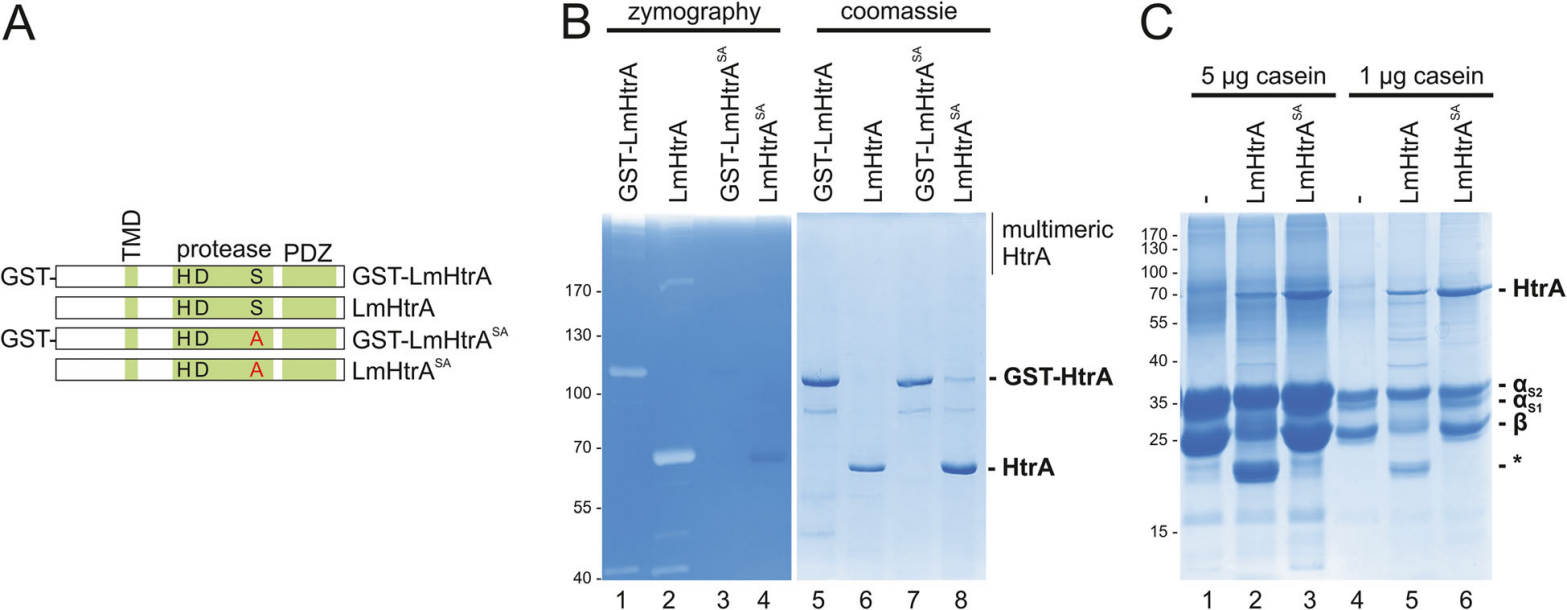
Cloning, overexpression and activity of *L. monocytogenes* HtrA. **a** HtrA consists of a transmembrane domain, a conserved serine protease domain containing the catalytic triad histidine (H229), aspartic acid (D259), and serine (S343) and a single PDZ domain as annotated by the MEROPS database. Expression constructs for N-terminally, removable GST-tagged HtrA were cloned. Serine 343 in the active center of HtrA was exchanged by an alanine (S343A) to create proteolytic inactive HtrA. **b** 200 ng of recombinant HtrA proteins were analyzed by casein zymography (left panel) and coomassie-stained protein gels (right panel). **c** 5 μg or 1 μg casein were incubated with 1 μg LmHtrA wt or LmHtrA^SA^ for 16 h at 37 °C. Samples were separated by SDS PAGE and proteins were stained with coomassie blue. Asterisks (*) label casein cleavage products. These assays have been performed as three independent experiments

The HtrA amino acid sequence of *L. monocytogenes* (UniProt Q8YA67) annotates an N-terminally located transmembrane domain (TMD), a catalytic triad composed of histidine (H), aspartic acid (D) and serine (S) in the protease domain and a single PDZ domain at the C-terminus (Fig. 2a). To analyze the proteolytic activity of LmHtrA, the *htra* gene from the EGD wildtype strain was cloned and overexpressed in *E. coli* to purify full length LmHtrA as an N-terminally tagged GST-LmHtrA fusion protein. To generate a proteolytic inactive HtrA protein, serine residue 343 in the active center of HtrA was replaced by an alanine (GST-LmHtrA^SA^). Finally, removal of the GST tag using the prescission protease resulted in untagged LmHtrA wildtype and LmHtrA^SA^ proteins (Fig. 2a). Purified recombinant proteins were then separated by a zymogram using casein as substrate, which has been previously established as an HtrA substrate [16, 17]. Both, GST-LmHtrA and LmHtrA migrated as full length monomers and formed multimeric structures in the zymogram, which were caseinolytically active. Additionally, truncated HtrA fragments were observed indicating autoprocessing of LmHtrA. Point mutation of serine 343 completely abrogated GST-LmHtrA^SA^ and LmHtrA^SA^ activity as neither casein degradation nor autoprocessing was observed (Fig. 2b, lanes 1–4). Purity and equal loading of recombinant proteins were examined in a coomassie-stained protein gel (Fig. 2b, lanes 5–8). The proteolytic activity was further analyzed in in vitro cleavage experiments. 5 μg (Fig. 2c, lanes 1–3) or 1 μg casein (Fig. 2c, lanes 4–6) were incubated with 1 μg LmHtrA or LmHtrA^SA^ followed by SDS PAGE and coomassie blue staining. Casein consists of α_S1_-, α_S2_-, and β-casein and was degraded by LmHtrA, but not by LmHtrA^SA^ (Fig. 2c), while β-casein was more efficiently targeted by LmHtrA than α_S2_-casein.

**Fig. 3:**
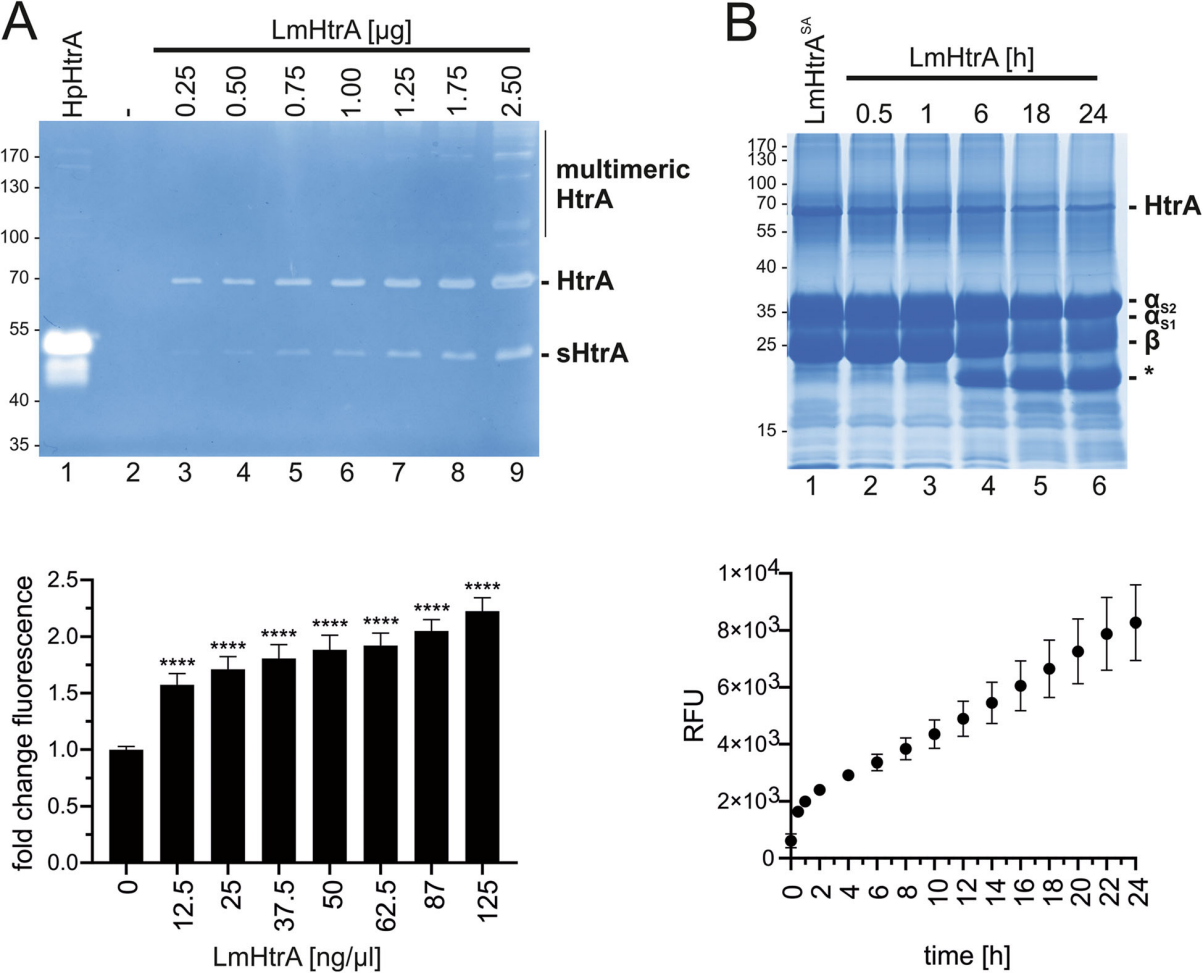
*L. monocytogenes* HtrA is highly active. **a** Indicated protein amounts of LmHtrA in 20 μl HEPES were analyzed by casein zymography. 3 μg *H. pylori* HtrA (HpHtrA) was used as a positive control. A truncated version of HtrA (short HtrA, sHtrA) has been detected (upper panel). Quantification of LmHtrA activity was performed in a fluorometric assay using FITC-casein as a substrate (lower panel) and indicated concentrations of LmHtrA, which corresponds to the used amounts of LmHtrA in the zymogram. These results are presented as fold change in fluorescence with untreated FITC-casein set to 1. **b** 5 μg casein were incubated with 1 μg LmHtrA for the indicated periods of time. LmHtrA^SA^ was used as a negative control. Cleavage activity was detected by coomassie staining of proteins (upper panel). Quantification of LmHtrA activity was performed in a fluorometric assay using FITC-casein (lower panel) and indicated concentrations of LmHtrA, which corresponds to the used amounts of LmHtrA in the zymogram. The kinetics of LmHtrA-mediated FITC-casein cleavage was analyzed and presented as relative fluorescent units (RFU). All data were obtained from three independent experiments with at least three technical replicates

To investigate the activity of recombinant LmHtrA, increasing amounts of LmHtrA have been analyzed by casein zymography. 0.25 μg LmHtrA was sufficient to detect caseinolytic activity (Fig. 3a, lane 3, upper panel). As a comparative control, 3 μg of recombinant *H. pylori* HtrA (HpHtrA) was included. Multimers of LmHtrA could be detected at protein amounts higher than 1.25 μg protein, which were more pronounced in comparison to HpHtrA multimers. Additionally, a truncated and active version of recombinant HtrA (short HtrA, sHtrA) was detected, which has been also observed for autoprocessed HtrA from other bacterial species previously [18] (Fig. 3a, upper panel). The LmHtrA activity was further investigated in a fluorometric assay using FITC-labeled casein as a substrate revealing a highly significant increase in casein cleavage (Fig. 3a, lower panel). The kinetics of LmHtrA activity were further investigated in in vitro cleavage experiments. Casein cleavage was observed after 6 h incubation at 37 °C and was almost complete after 18 h and 24 h (Fig. 3b, upper panel) pointing to a strong activity of LmHtrA. This has been confirmed by the fluorometric casein assay (Fig. 3b, lower panel).

**Fig. 4:**
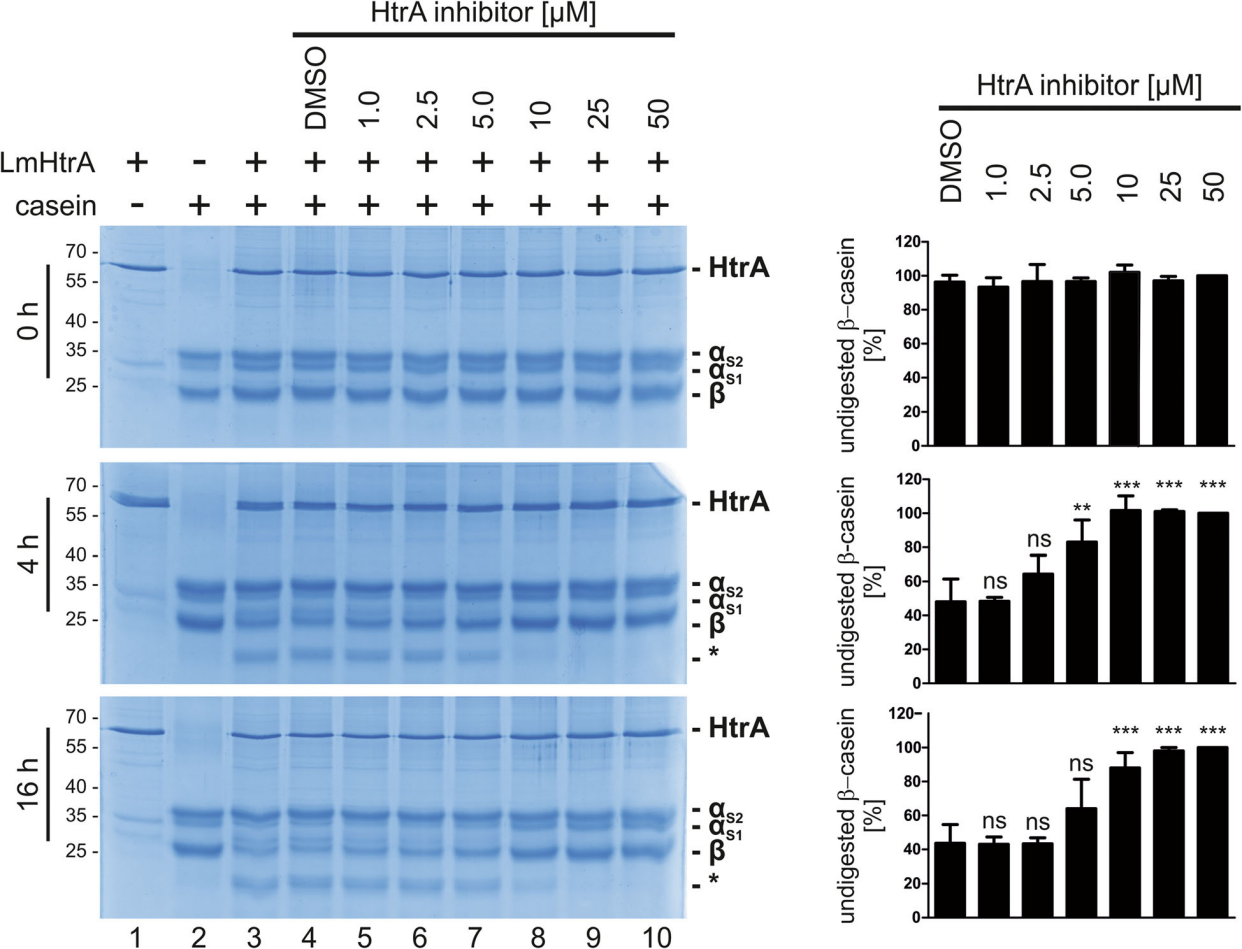
Inhibition of LmHtrA. 2 μg of recombinant LmHtrA were incubated with 10 μg casein and increasing concentrations (1–50 μM) of a small molecular HtrA inhibitor. The relative amount of β-casein was quantified by densitometry from three independent experiments. These results are given as arbitrary units with β-casein levels which were treated with 50 μM inhibitor set to 100%. Asterisks (*) label casein cleavage products

Since HtrA proteases were described as pathogenicity-relevant bacterial factors, several potent small molecule inhibitors have been developed to block bacterial HtrA proteases [19, 20]. The small molecule (compound 1a, [20]) slightly inhibited LmHtrA at a concentration of 1 μM in in vitro casein cleavage experiments. Complete inhibition of LmHtrA activity was seen using a concentration of 10 μM HtrA inhibitor after 4 h and 16 h cleavage (Fig. 4, left panel). Densitometric analyses of three independent experiments underlined observed inhibition of LmHtrA (Fig. 4, right panel) indicating that LmHtrA activity represents a druggable target.

**Fig. 5:**
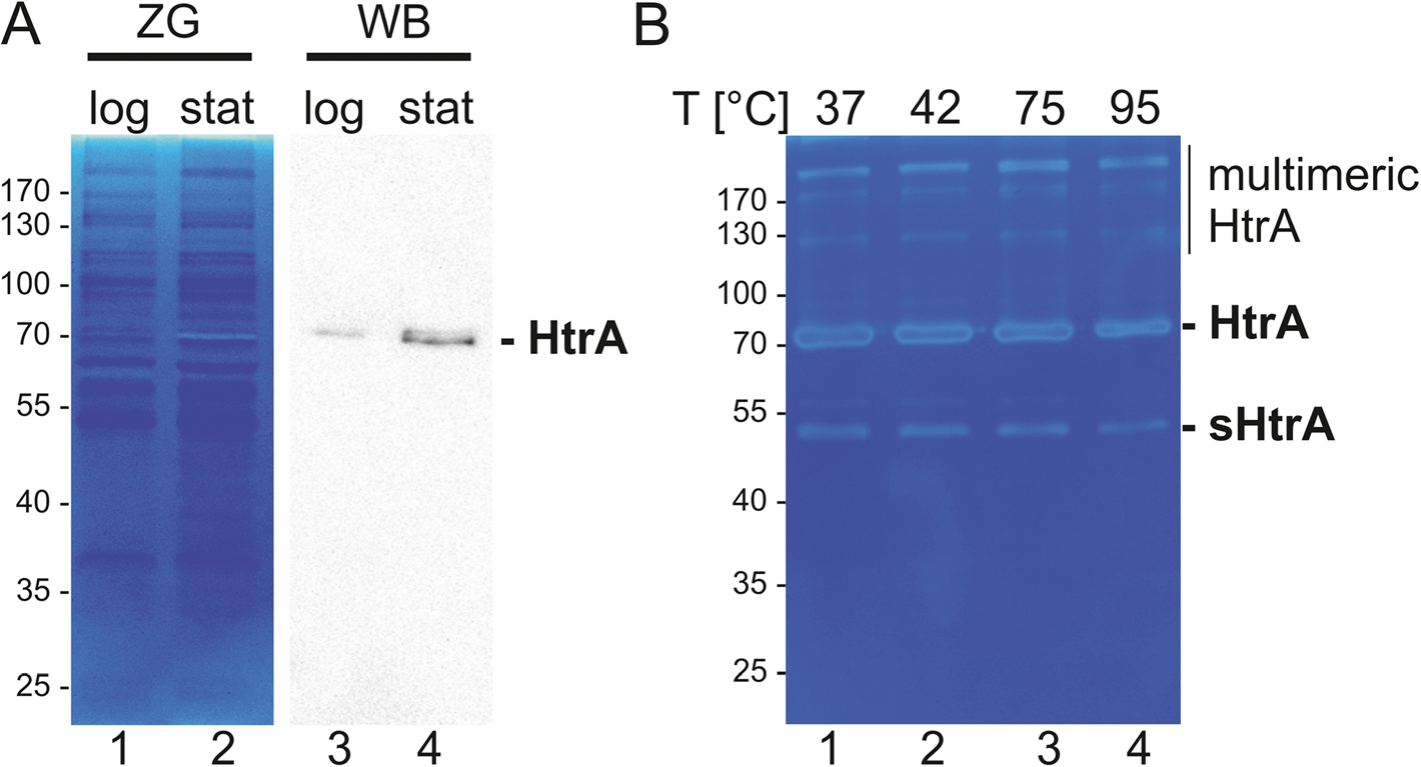
*L. monocytogenes* HtrA is active under stress conditions. **a** The activity of LmHtrA expressed by *L. monocytogenes* EGD grown to the logarithmic phase or stationary phase was detected in 50 μg whole cell lysates by casein zymography (left panel). 10 μg whole cell lysate of *L. monocytogenes* EGD in different growth phases were analyzed for HtrA expression by Western blotting using a specific anti-LmHtrA antibody (right panel). The zymogram has been repeated four times with lysates from independent experiment and the HtrA detection by Western blot has been performed by two independent experiments. **b** Recombinant LmHtrA was incubated at indicated temperatures for 30 min and the activity of HtrA multimers, monomers and truncated HtrA (short HtrA, sHtrA) was investigated in casein zymography. This experiment has been repeated three times

**Fig. 6:**
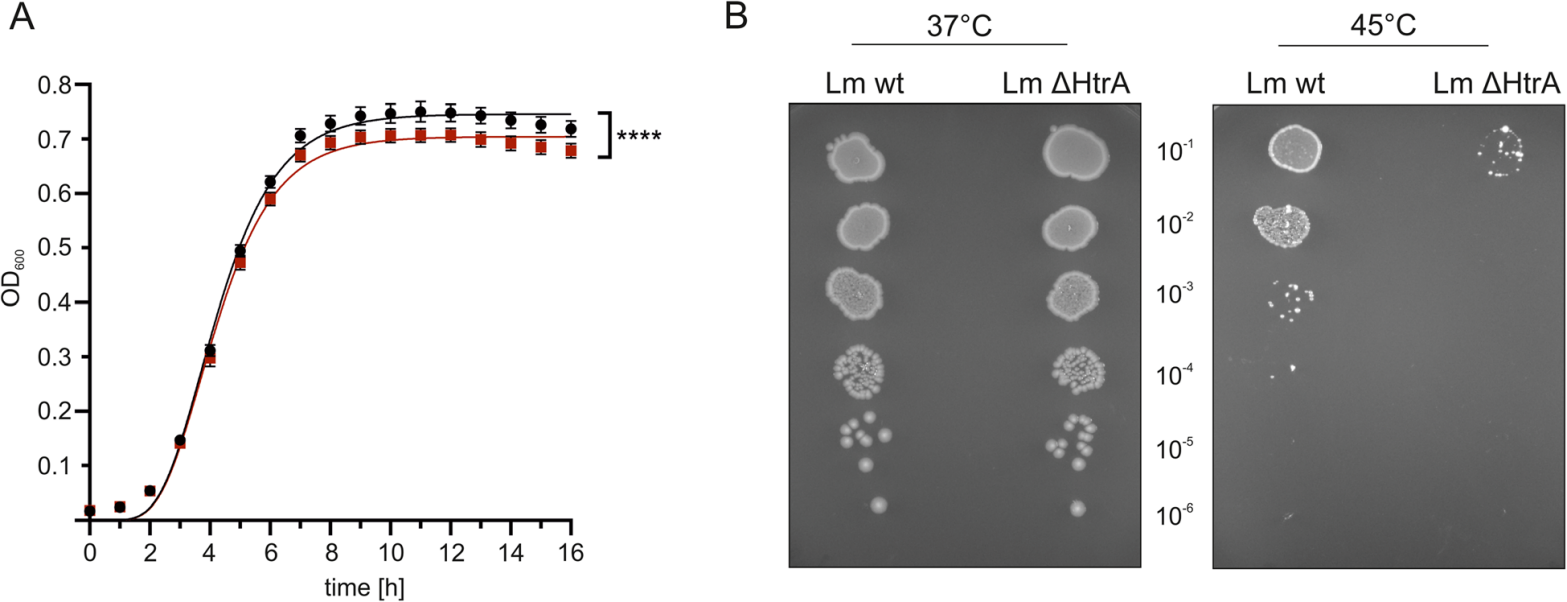
*L. monocytogenes* HtrA enhances bacterial survival. **a** Real-time growth of *L. monocytogenes* 10403 s wildtype (black circles) and *L. monocytogenes Δhtra* (red squares) was monitored at 37 °C by measuring the OD_600_. This experiment has been repeated two times with octaplicates as technical replicates. Statistical analysis revealed F(DFn, DFd) = 110.1 (3, 538) and *p* < 0.0001. F and *p* value lead to rejection of H_0_ and indicated a significant difference between the growth curves. **b** 10-fold serial dilutions of exponentially growing cultures were spotted on BHI agar plates and incubated at 37 °C and 45 °C. This experiment has been repeated three times

In a previous study we detected unknown caseinolytic activities exhibiting a molecular weight of 70 kDa and > 170 kDa which were upregulated in the stationary growth phase of *L. monocytogenes* [21]. In this report, we identified active LmHtrA as the responsible protease with the molecular weight of 70 kDa for the monomer and > 170 kDa for the multimers. Hence, we analyzed the expression of LmHtrA in the logarithmic and stationary growth phase of *L. monocytogenes* and observed both, an increase of the caseinolytic active LmHtrA monomer and multimer in bacteria grown to the stationary growth phase in zymography and Western blot analysis (Fig. 5a). These data underline the hypothesis that HtrA is important for bacteria under starvation and stress conditions. This was further supported by the finding that recombinant LmHtrA tolerated extreme temperatures. LmHtrA was incubated at indicated temperatures for 30 min and its activity was subsequently investigated in casein zymography. These experiments revealed that LmHtrA and the truncated sHtrA resisted high temperatures up to 95 °C and can still be detected as an active protease in casein zymography (Fig. 5b). The benefit of HtrA expression for listerial growth was further examined by real-time growth monitoring (Fig. 6a) and colony assays at elevated temperatures (Fig. 6b). At 37 °C, the growth of *L. monocytogenes* wildtype significantly differed from the growth of *L. monocytogenes ΔhtrA*. After 6 h incubation, the liquid culture of *L. monocytogenes* wildtype showed slightly enhanced density, which became more obvious after 8 h to 16 h growth (Fig. 6a). This was confirmed by the colony assay. No obvious effects between *L. monocytogenes* wildtype and *L. monocytogenes ΔhtrA* were observed after the culture at 37 °C (Fig. 6b, left panel). However, increasing the temperature to 45 °C led to a drastic loss of survival of *L. monocytogenes* wildtype, which was more pronounced for *L. monocytogenes ΔhtrA* (Fig. 6b, right panel) underlining the beneficial effect of HtrA expression in *L. monocytogenes* growth and fitness under stress conditions.

## Discussion

The serine protease and chaperone HtrA has consistently been described as a crucially important factor in promoting bacterial fitness and survival under stress conditions. In this context, its chaperone function mainly promotes folding and assembly of unfolded proteins, whereas the protease function removes misfolded proteins to prevent their toxic accumulation [22]. In the foodborne pathogen *L. monocytogenes* similar functions have been suggested since several research groups reported that the expression of HtrA in *L. monocytogenes* represents a significant contribution to bacterial growth and survival under stress conditions and to in vivo pathogenesis [5–8]. However, the expression and protease activity of listerial HtrA has never been investigated. In this study, we performed a detailed analysis of LmHtrA and demonstrated the robust proteolytic LmHtrA activity in *L. monocytogenes*, efficient inhibition of LmHtrA using a small molecule HtrA inhibitor, and altered LmHtrA expression levels at different growth phases.

The activity of HtrA proteases depends on the formation of oligomeric structures built by trimers. It has been described that substrate binding to the PDZ domain of DegP or DegQ induces oligomer conversion from inactive hexameric into proteolytically active 12-mers and 24-mers [23, 24]. In another study it was shown that trimeric DegP can still degrade substrates [25]. Those studies were mainly performed in Gram-negative bacteria. The HtrA activity in Gram-positive bacteria is largely unknown and requires more intensive research because the domain architecture differs considerably. In our study, we detected activities derived from LmHtrA forms migrating at the molecular weights of monomers and oligomers in zymography, which is consistent with observations made in *H. pylori*, *Campylobacter jejuni* or *E. coli* [16, 26]. Formation of oligomeric structures was still possible after extensive exposure to high temperature indicating that LmHtrA is highly stable and can refold into functional proteases during zymography. Whether HtrA proteins form active oligomers in the renatured gel or whether the monomeric HtrA is activated under these conditions in a zymogram needs to be investigated in future studies. In addition, we detected a truncated LmHtrA (sHtrA) version, which has also been described in Gram-negative bacteria, such as *E. coli* and *H. pylori* [10, 16, 17, 27]. In *H. pylori*, N-terminal cleavage of HtrA is accompanied with a decrease of trimer formation and HtrA activity [28, 29]. This appears to be in contrast to HtrA proteases expressed by Gram-positive bacteria. In *Bacillus anthracis*, N-terminal autoprocessing has been demonstrated as a requirement for both, secretion and activity [18]. Although it is not known whether truncated sHtrA from *L. monocytogenes* is the result of N-terminal or C-terminal cleavage, truncated LmHtrA is still caseinolytically active suggesting that *L. monocytogenes* also secretes HtrA into the environment as an active protease.

Efficient proteolytic removal of misfolded and/or damaged proteins is essential for intracellular protein-quality control. Starved *L. monocytogenes* grown to the stationary phase upregulated the expression of LmHtrA as shown in zymography and Western blot analyses. These data underline the importance of LmHtrA in the maintenance of bacterial fitness under stress conditions and biofilm formation. The expression of LmHtrA has previously been associated with the ability to develop listerial biofilms [7]. Together with the findings that LmHtrA is highly stable at high temperatures and significantly contributes to bacterial growth at elevated temperature, we confirm that LmHtrA expression and activity support bacterial fitness. As a foodborne pathogen, this phenotype essentially increases the transmission of *L. monocytogenes* with food, but also promotes listerial pathogenesis through a better tolerance against stress during infection. In our study, we found that a small molecule HtrA inhibitor potently inhibited LmHtrA.

## Conclusions

Deletion of *htrA* in the genome of *L. monocytogenes* induces a severe defect in listerial pathogenesis. In this study we demonstrated a robust proteolytic activity of LmHtrA responsible for enhanced growth and survival under stress conditions. Since previous studies also indicated an interference of LmHtrA expression and the antibiotics puromycin and penicillin G [6, 7], LmHtrA inhibition might represent an attractive additive to the treatment of listeriosis with antibiotics.

## Methods

### Bacteria

*Listeria monocytogenes* 10403 s wildtype and its isogenic *Δhtra* deletion mutant were obtained from Rebecca Wilson (SIGA Technologies, Corvallis, USA) and *L. monocytogenes* EGD was received from Pascale Cossart (Institut Pasteur, Paris, France) and have been previously described [7, 30]. For the measurement of bacterial growth, bacteria were cultivated in brain heart fusion (BHI) medium at 37 °C. The medium for *L. monocytogenes ΔhtrA* was supplemented with 50 μg/ml streptomycin. Bacterial overnight cultures were diluted to an OD_600_ of 0.01. 200 μl bacterial suspension was then transferred to transparent Nunclon™ Edge 96-well plates (SIFIN GmbH, Berlin, Germany) and shaken for 24 h at 37 °C in a M200 PRO Quad4 Monochromators™ -based multimode reader (Tecan, Anif, Austria). The plate moats were loaded with 4 × 3 ml of 0.1% agarose to reduce evaporation of the culture medium. Growth was monitored by detecting the optical density at λ = 600 every 30 min. Assays were performed as octaplicates for *L. monocytogenes* wildtype and *L. monocytogenes ΔhtrA*. For the colony assay, overnight cultures were diluted to an OD_600_ of 0.05 and grown to an OD_600_ of 0.3 at 37 °C and 200 rpm. Four μl of 10-fold serial dilutions of exponentially growing cultures were spotted on BHI agar plates and incubated at 37 °C or 45 °C for 48 h.

### Cloning, mutagenesis and purification of HtrA

LmHtrA (lmo0292, gene ID 987455) was amplified from genomic DNA of the *L. monocytogenes* strain EGD. The amplified BamHI/EcoRI flanked PCR product was cloned into the pGEX-6P-1 plasmid (GE Healthcare Life Sciences) and transformed into *E. coli* BL21 to express a GST-HtrA fusion protein. To generate a protease-inactive HtrA protein, serine 343 was substituted by an alanine (HtrA^SA^) using the QuikChange Lightning Site-Directed Mutagenesis Kit (Agilent) according to the manufacturer’s instructions. For heterologous expression and purification of HtrA proteins, transformed *E. coli* was grown in 300 ml LB medium to an OD_600_ of 0.5–0.7 at 37 °C and 200 rpm and the expression was induced by the addition of 0.1 mM Isopropyl β-D-1-thiogalactopyranoside (IPTG) at 30 °C for 3 h. The bacterial culture was pelleted at 4500 x g for 30 min and bacteria were lysed in 10 ml ice-cold PBS by sonication on ice 3 times for 45 s with 50% power (Sonoplus Ultraschall Homogenisator HD270, Bandelin electronic GmbH, Berlin, Germany). The lysate was cleared by centrifugation and the supernatant was incubated with glutathione sepharose (GE Healthcare Life Sciences, Vienna, Austria) at 4 °C overnight as described earlier [16]. The on-column fusion protein was either eluted with 10 mM reduced glutathione for 10 min at room temperature or cleaved with 180 U prescission protease (2 U/100 μg GST-LmHtrA) for 16 h at 4 °C (GE Healthcare Life Sciences, Vienna, Austria) to obtain an untagged HtrA protein. Purified proteins were rebuffered in 50 mM HEPES, pH 7.4. The activity and purity of recombinant proteins have been routinely tested by zymography and coomassie-stained SDS PAGEs.

### SDS PAGE and Western blot

Lysates of *L. monocytogenes* were prepared as described before [21]. 10 μg of lysates was separated by SDS-PAGE under reducing conditions and stained using 1% Coomassie Brilliant Blue G250 (BioRad, Vienna, Austria). For Western blot analyses, 10 μg of proteins were separated by SDS-PAGE and blotted on PVDF membranes. For LmHtrA, 10% gels and for casein detection, 12% gels were used. LmHtrA was detected using a polyclonal anti-LmHtrA antiserum produced in rabbits immunized with recombinant LmHtrA^SA^ (Davids Biotechnology, Regensburg, Germany). Visualizing was performed using Odyssey 1 Fc Imaging System (LiCor, Bad Homburg Germany).

### In vitro cleavage assays

For in vitro cleavage assays, 1–10 μg of casein (Lactan, Graz, Austria) was incubated with 1–2 μg LmHtrA in 50 μl of 50 mM HEPES, pH 7.4 for 16 h or indicated time periods at 37 °C. The proteins were separated using SDS-PAGE and casein cleavage was visualized using Coomassie G250 (Lactan, Graz, Austria). Where indicated a small molecule HtrA inhibitor (compound 1a [20]) was added to block LmHtrA activity.

### Zymography

Bacterial cultures were centrifuged at 4500×g for 20 min at 4 °C. Pelleted bacteria were then harvested in lysis buffer (20 mM Tris pH 7.5, 100 mM NaCl, 1% Triton X-100, 0.5% deoxycholate [DOC], 0.1% SDS, 0.5% NP-40). Lysates were sonicated on ice 3 times for 45 s with 50% power and were then centrifuged at 16000×g for 20 min at 4 °C. Bacterial lysates or recombinant proteins were separated by SDS-PAGE containing 0.1% casein under non-reducing conditions as described previously [16]. Proteins separated by the gels were renatured in 2.5% Triton X-100 for 1 h, incubated in developing buffer (50 mM Tris pH 7.5, 200 mM NaCl, 5 mM CaCl_2_, 0.02% detergent Brij35) for 16 h at 37 °C and stained using 0.5% Coomassie Brilliant Blue R250 (Lactan, Graz, Austria).

### Protease activity assay using FITC-labeled casein

Quantification of LmHtrA activity was performed using a fluorescent protease assay kit (Thermo Scientific, Vienna, Austria). The measurements were performed in a white, flat bottom 96-well plate (Nunc) at 37 °C. 5 ng/μl FITC-casein was incubated with indicated concentrations of LmHtrA and the fluorescence was measured in a plate reader (Infinite® 200 PRO, TECAN, Anif, Austria) with a filter setting of 485 nm/535 nm (Ex/Em).

### Statistics

All statistical analyses were performed with GraphPad Prism software version 8.0.2. The Gompertz model was used to describe and statistically compare the bacterial growth of *Lm* wt and *Lm* ΔHtrA [31, 32]. For comparison of the fitted curves, the extra sum-of-squares F-Test and unpaired t-test was used with the null hypothesis (H_0_): one curve for all datasets and alternative hypothesis (H_1_): different curves for all datasets. Quantification of β-casein cleavage as shown in Fig. 4 was done by measuring integrated band intensities using ImageLab (BioRad, Vienna, Austria). All values were normalized to the intensity of β-casein band at 50 μM (set as 100%). Statistical evaluations between control (DMSO) and different inhibitor concentrations were calculated using ANOVA and significance was tested using Tukey post hoc test. For the statistical analysis of the FITC-casein assay means were normalized to uncleaved FITC-casein. One-way ANOVA as used to statistically compare the fold change of fluorescence between samples treated with increasing LmHtrA and the non-treated control. Significance was tested using the Dunnett test. Three independent experiments containing three technical replicates were analyzed. Significance indicated as non-specific (ns) > 0.05, * for *p* < 0.05, ** for *p* < 0.01, *** for *p* < 0.001, and **** for *p* < 0.0001.

## Data Availability

The datasets supporting the conclusions of this article are included within the article and its additional files.

## Abbreviations

HtrA: high temperature requirement A
*L. monocytogenes*: *Listeria monocytogenes*
